# A randomized physiotherapy trial in patients with fecal incontinence: design of the PhysioFIT-study

**DOI:** 10.1186/1471-2458-7-355

**Published:** 2007-12-20

**Authors:** Esther MJ Bols, Bary CM Berghmans, Erik JM Hendriks, Rob A de Bie, Jarno Melenhorst, Wim G van Gemert, Cor GMI Baeten

**Affiliations:** 1 Department of Epidemiology, Maastricht University, PO Box 616, 6200 MD Maastricht, The Netherlands; 2 Caphri research institute, Maastricht University, The Netherlands; 3 Pelvic care Center Maastricht, University Hospital Maastricht, PO Box 5800, 6202 AZ Maastricht, The Netherlands; 4 Centre for Evidence Based Physiotherapy, Maastricht University, PO Box 616, 6200 MD Maastricht, The Netherlands; 5 Department of Surgery, University Hospital Maastricht, PO Box 5800, 6202 AZ Maastricht, The Netherlands

## Abstract

**Background:**

Fecal incontinence (FI) is defined as the recurrent involuntary excretion of feces in inappropriate places or at inappropriate times. It is a major and highly embarrassing health care problem which affects about 2 to 24% of the adult population. The prevalence increases with age in both men and women.

Physiotherapy interventions are often considered a first-line approach due to its safe and non-invasive nature when dietary and pharmaceutical treatment fails or in addition to this treatment regime. Two physiotherapy interventions, rectal balloon training (RBT) and pelvic floor muscle training (PFMT) are widely used in the management of FI. However, their effectiveness remains uncertain since well-designed trials on the effectiveness of RBT and PFMT versus PFMT alone in FI have never been published.

**Methods/Design:**

A two-armed randomized controlled clinical trial will be conducted. One hundred and six patients are randomized to receive either PFMT combined with RBT or PFMT alone. Physicians in the University Hospital Maastricht include eligible participants. Inclusion criteria are (1) adults (aged ≥ 18 years), (2) with fecal incontinence complaints due to different etiologies persisting for at least six months, (3) having a Vaizey incontinence score of at least 12, (4) and failure of conservative treatment (including dietary adaptations and pharmacological agents). Baseline measurements consist of the Vaizey incontinence score, medical history, physical examination, medication use, anorectal manometry, rectal capacity measurement, anorectal sensation, anal endosonography, defecography, symptom diary, Fecal Incontinence Quality of Life scale (FIQL) and the PREFAB-score. Follow-up measurements are scheduled at three, six and 12 months after inclusion. Skilled and registered physiotherapists experienced in women's health perform physiotherapy treatment. Twelve sessions are administered during three months according to a standardized protocol.

**Discussion:**

This section discusses the decision to publish a trial protocol, the actions taken to minimize bias and confounding in the design, explains the choice for two treatment groups, discusses the secondary goals of this study and indicates the impact of this trial on clinical practice.

**Trial registration:**

The Netherlands Trial Register ISRCTN78640169.

## Background

### Background of fecal incontinence

Fecal incontinence (FI) is a major health care problem, that is particularly embarrassing and affects about 2 to 24% of the adult population, increasing to 47% in the institutionalized elderly [1-7]. The actual prevalence is likely to be higher due to the common underreporting of FI as a result of patients' embarrassment to visit a physician or unawareness of possible treatment options [8]. FI can be defined as the recurrent involuntary excretion of feces in inappropriate places or at inappropriate times [7], and covers a wide spectrum from involuntary but recognized passage of gas, liquid, or solid stool (urge incontinence) to unrecognized anal leakage of mucus, fluid, or stool (passive incontinence) [9]. Fecal continence is based on a combined interplay of sensory, motoric and reservoir functions. Incontinence occurs if one or more of these components fail and when compensatory mechanisms fall short. Obstetric trauma is one of the major causes of FI in women. Several colorectal, urological or gynaecological interventions can cause FI as well. Specific neurological diseases associated with FI include diabetes, multiple sclerosis, Parkinson's disease, stroke, and spinal cord injury.

FI is often thought to be associated with older people, as a natural aspect of ageing. Recently, it has been estimated that 6% of the persons aged 60 years and older suffer from involuntary loss of feces and 3% from involuntary loss of both feces and urine [8]. In addition, the prevalence of FI will increase in the next fifteen years due to double ageing. However, younger patients are often affected as well [1]. FI interacts with multiple aspects of daily life resulting in a number of difficulties; having to stay at home near a toilet, having to avoid social contacts including relationships or sexual contact, having feelings of depression and low self-esteem [2]. Moreover, it is often difficult to explain experiencing FI to others, like family or a partner. A lot of these implications are due to the unpredictable character of FI and the fear of odour or soiling [10].

Apart from a social burden to the patient, FI also has a significant impact on the health care budget. Limited data are available on healthcare costs for FI [9,11]. Recently, total costs in the Netherlands were estimated at € 2169 per fecally incontinent patient a year [12].

Estimates of health care costs and prevalence numbers for FI vary greatly and depend on the clinical setting and on the definition of FI based on severity and frequency [1,13,14].

### Physiotherapy in fecal incontinence

Treatment of fecally incontinent patients consists of conservative as well as surgical interventions. Surgical interventions aim to anatomically and functionally correct the rectum, anus, pelvic floor or anal sphincters. Conservative interventions consist of dietary adaptations, medication, absorbent materials, and physiotherapy. Physiotherapy interventions are non-invasive and inexpensive, require no sophisticated equipment and have hardly any adverse effects. Besides, physiotherapy does not exclude any other form of treatment [15]. Therefore, physiotherapy is often attempted before surgical treatment.

Physiotherapy FI management includes pelvic floor muscle training (PFMT), biofeedback (BF), rectal balloon training (RBT) and electrical stimulation (ES). PFMT aims to restore muscular strength, coordination and timing of contractions. With BF the patient gets information about the activity of the pelvic floor muscles by way of a visual display. RBT, as a modality of BF, is used to decrease the sensory threshold in case of passive FI and to increase the sensory threshold in case of urge FI. In addition, RBT aims to synchronize contraction of the external anal sphincter with relaxation of the internal anal sphincter. ES is used to increase awareness and isolated contraction of the anal sphincters. Often, one or more physiotherapy interventions are combined, depending on the underlying cause of FI.

Over 60 uncontrolled trials exist on the use of biofeedback for the management of FI [16]. Some authors conclude that biofeedback is the treatment of choice for FI on the basis of these observational studies [17]. An overall cure and improvement rate of 72 percent has been reported [18]. However, the results of a Cochrane review on the effects of BF and/or PFMT for the treatment of FI in adults were based on eleven randomized controlled but heterogeneous trials and showed that some elements of BF therapy and sphincter exercises might have a therapeutic effect, but this is not certain [16]. Moreover, it was suggested that RBT improved continence more than sham training. This is in agreement with other authors who consider lowering the threshold volume for discrimination of rectal distension an important factor in the success of BF [15,19-21].

In an attempt to assess the effect of the different BF components (balloon/pressure BF, electromyographic BF) in the management of FI, two complicated cross-over trials have been performed [15,20]. Group comparison was impossible to assess due to the small sample in one trial (16) and the single case experiments in the other (21).

A second Cochrane review evaluated ES in adult patients with FI [16,22]. Insufficient data was available to allow reliable conclusions on the effect of ES in the management of FI [22]. A recently published randomized controlled trial examined whether anal ES, in the absence of any adjunct exercises or advice, would improve symptoms more when compared with "sham" ES. It is concluded that 1 Hz (sham) was as effective as 35 Hz. Possibly, the main effect is not sphincter contraction but sensitization of the patient to the anal area, or simply the effect of intervening per se (Hawthorne effect) [23].

Finally, the results of a recent large cohort study on ES and PFMT with BF in patients with severe FI indicated that pelvic floor muscle rehabilitation leads to a modest improvement in severity of FI, squeeze pressure and maximal tolerated volume [24].

Although uncertainty exists on the exact extent of the effectiveness of physiotherapy treatment in FI, results are promising [18].

### Relevance

Evaluating effectiveness of physiotherapy treatment has been impeded by lack of enough quality studies, lack of standardization, a large variety in outcome measures and methodological flaws [25]. Ultimately, there is a need to evaluate treatment options in FI, especially since FI considerably reduces quality of life. Besides, FI has a significant impact on the health care budgets, and the prevalence of FI is increasing due to double ageing [26].

PFMT has been proven effective in the treatment of stress urinary incontinence [27]. Since the anal sphincter and puborectalis muscle form a part of the same pelvic floor as the closing urethral system, expectations are raised for the same positive results of pelvic floor muscle re-educative techniques in FI. Next to PFMT, some authors consider lowering the threshold volume for discrimination of rectal distension as an important factor in the success of physiotherapy [15,19-21]. No study has reported adverse events from either PFMT or RBT, and it seems unlikely that these treatments itself could cause symptoms to worsen.

Apparently, PFMT and RBT seem to have a role in the treatment of fecally incontinent patients. Since well-designed trials on the effectiveness of RBT and PFMT versus PFMT alone in patients with FI have never been published, a randomized controlled trial on this topic will be carried out (Physiotherapy in Fecal Incontinence Trial – PhysioFIT-study).

## Methods/Design

### Study design, research question and hypothesis

**Figure 1:**
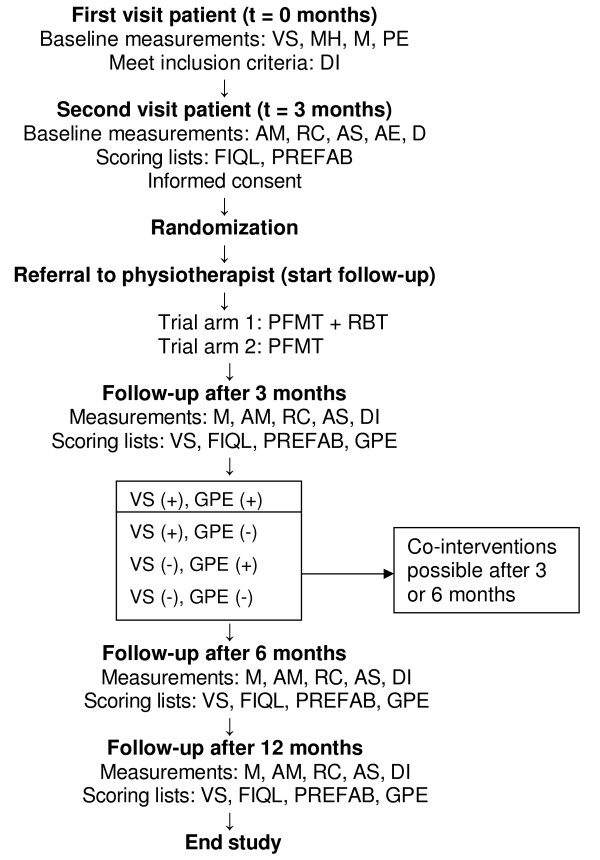
**Flowchart of the PhysioFIT-study**. t = point in time; VS = Vaizey score; MH = medical history; M = medication use ; PE = physical examination; DI = diary; AM = anorectal manometry; RC = rectal capacity measurement; AS = anorectal sensation; AE = anal endosonography; D = defecography; FIQL = fecal incontinence quality of life scale; PREFAB = modified PRAFAB-score; GPE = Global Perceived Effect-score; PFMT = pelvic floor muscle training; RBT = rectal balloon training; VS (-) = reduction on VS < 4 points; VS (+) = reduction on VS ≥ 4 points; GPE (+) = score 1–7 on GPE scale; GPE (-) = score 8 or 9 on GPE scale

A two-armed randomized controlled clinical trial will be conducted in patients diagnosed with FI (Figure 1), serving the following research question: what is the effectiveness of rectal balloon training and pelvic floor muscle training compared to pelvic floor muscle training alone in patients with fecal incontinence?

It is hypothesized that patients receiving the combined training will have a larger reduction on the primary outcome measure.

### Study population

The study population consists of patients living in the Netherlands. Participants are eligible when they are diagnosed with FI after general diagnostics. The randomized controlled trial is carried out at the University Hospital Maastricht, where two colorectal surgeons (CB, WG) include eligible participants. Inclusion criteria are (1) adults (aged ≥ 18 years), (2) FI complaints due to different etiologies persisting for at least six months, (3) Vaizey incontinence score of at least 12 [28], (4) and failure of conservative treatment (including dietary adaptations and pharmacological agents). Inclusion criteria 2, 3 and 4 indicate that patients with moderate to severe FI complaints are of interest. Patients diagnosed with an anorectal tumor within the past two years, absent squeeze pressure of the anal sphincter, chronic diarrhea (always fluid stool three or more times a day), overflow incontinence, proctitis, colitis ulcerosa, Crohn's disease, soiling (defined as leakage of a minimal amount of feces out of the anal canal), previous ileo-anal or colo-anal anastomosis and/or rectal prolapse in situ are excluded. Participants who received physiotherapy during the previous six months or who are intellectually and/or linguistically incapable to understand therapy are excluded as well.

### Recruitment, informed consent and randomization

The medical ethics committee of the University Hospital Maastricht/Maastricht University approved the PhysioFIT-study. The physician points out during the first visit that the patient is eligible to participate in the trial. The patient is provided with patient information sheets and information on the diagnostic tests. Participating patients sign informed consent at the beginning of the second visit, allowing eligible participants two months to decide to participate.

An independent research assistant performed the blocked randomization (size of four). A computer-generated randomization list is prepared in advance. The independent research assistant contacts the physiotherapist by telephone to inform about the referral and the allocated treatment.

### Blinding

The research doctor, performing the general diagnostics, and the researcher, dealing with the data-entry, are blinded during baseline measurements but not during follow-up measurements. However, the physician involved in measuring the primary outcome measure is blinded and blinded double data-entry is performed to assure correct data-entry. It is impossible to blind patients and participating physiotherapists for treatment allocation. However, physiotherapists are not involved in the diagnostic work-up and follow-up measurements. The outcome assessors are blinded.

### Baseline and follow-up measurements

Given the multiple etiologies in patients with FI, no test should be expected to yield consistent results in all patients with FI. Therefore, no standard protocol is available for the assessment and diagnosis of FI. The chosen tests at baseline (except for the questionnaires) are recommended by the American Gastroenterological Association, being procedures of value (symptom diary, physical examination, anal endosonography, anorectal manometry, rectal and anal sensory testing) and being procedures of possible value (defecography) [29]. As these diagnostic tests are part of the standard diagnostic assessment in the University Hospital Maastricht, participation in the trial will not burden the patient additionally. The following baseline measurements are done during the first and second patient visit:

▪ Vaizey incontinence score: this score reflects the severity of FI and ranges from 0 (complete continence) to 24 (complete incontinence) (Table 1) [28]. The Vaizey score is a widely used severity score and it is reported to be a reproducible score, which correlates highly with physicians' clinical impression [28]. Recently, it has also been demonstrated that higher Vaizey scores are associated with more reported problems in general health domains [30] and that changes in Vaizey score reflect patients' subjective perception of relief [31].

**Table 1:** Fecal incontinence score according to Vaizey et al. [28]

Incontinent	Never	Rarely	Sometimes	Weekly	Daily
Solid stool	0	1	2	3	4
Liquid stool	0	1	2	3	4
Gas	0	1	2	3	4
Alteration in lifestyle	0	1	2	3	4
					
				No	Yes
Need to wear a pad or plug				0	2
Taking constipating medication				0	2
Lack of ability to infer defecation for 15 minutes				0	4

Minimum score = 0 = perfect continence; maximum score = 24 = totally incontinent Never: no episodes in the past 4 weeks; rarely: one episode in the past 4 weeks; sometimes: > 1 episode in the past 4 weeks, but < once a week; weekly: one or more episodes a week but < one a day; daily: one or more episodes a day

▪ Medical history: the medical history assesses gender, age, duration of complaints, risk factors (surgical, obstetric, neurological, metabolic) and medication use.

▪ Physical examination: digital rectal examination is used to assess anal pressure at rest and pressure during contraction. Sphincter defects can be located and palpated, especially during contraction. In women, the presence of a rectocele can be established.

▪ Anorectal manometry: anorectal manometry is performed to measure anal sphincter pressures. The test is performed in the left lateral position and flexed hips to 90 degrees. Anal manometry takes place according to the water-perfused technique. The single use manometric catheter (Medtronic Inc., Skovlunde, Denmark) is introduced, which is connected to a computer-assisted polygraph (Polygraf™ ID, Medtronic Inc., Skovlunde, Denmark) and stabilized in the anal sphincter complex. After positioning the catheter in the maximal pressure area, the basal sphincter pressure, maximum squeeze pressure and rectal anal inhibitory reflex are measured.

▪ Rectal capacity measurement: the measurement is performed by introducing a single use manometric catheter (Medtronic Inc., Skovlunde, Denmark) in the rectum, which is connected to a computer-assisted polygraph (Polygraf™ ID, Medtronic Inc., Skovlunde, Denmark). The catheter is inflated with air with slow increments. The volumes are recorded at which first sensation is noticed (sensory threshold), an urge to evacuate is produced (urge sensation), and volume at which pain becomes intolerable (maximum tolerated volume).

▪ Anorectal sensation: a St. Mark's glove pudendus electrode is placed on the index finger and introduced into the anal canal. An increasing electrical current (mAmp) is gradually applied until the threshold of sensation is reached. Anorectal sensation is measured at two places: three o'clock and nine o'clock in the transversal plane.

▪ Anal endosonography: anal endosonography provides an anatomical image of the anal sphincters and pelvic floor. This technique gives an instant excellent image of the condition of the sphincters and establishes the size of a possible internal or external anal sphincter defect. Anal endosonography is performed with an ultrasound scanner with radial endoscopic probe and a 7.5-MHz transducer. The probe is covered with a lubricant and a condom. After application of a final lubricant the probe is introduced in the anal canal with the patient in the left lateral or prone position. The probe is slightly withdrawn, so all the different levels of the anal sphincter complex can be visualized.

▪ Defecography: defecography is a dynamic radiological study of attempted defecation and shows both the functional aspects of defecation and defects in anorectal anatomy. Patients are instructed to drink contrast medium diluted water before examination. The test starts with the patient in left decubital position. Through an injection pistol barium paste is manually injected in the rectum. In female patients gel is also injected via a syringe in the vagina. The perineum is located with a gel solution as well. Subsequently, the entire x-ray table is tilted upright 90° and the patient is seated on a specially developed radiolucent defecography chair. After the test the patient is instructed to drink sufficiently to eliminate the contrast.

▪ Diary: the diary gives insight into the defecation pattern, involuntary incontinence episodes, pad use, presence of urge feelings, presence of urinary incontinence, alteration in daily activities, ability to infer defecation and medication use of a patient. The diary monitors three weeks. Since FI often goes together with urinary incontinence, additional questions will be included concerning the severity of urinary incontinence. Since limited information is available about the quantity of absorbent material use and the associated costs, the diary will enclose additional questions to get more insights into this domain and estimate accompanying health care costs.

▪ Fecal incontinence quality of life scale (FIQL): the FIQL is a disease-specific quality of life scale, comprising four scales: lifestyle (10 items), coping/behavior (9 items), depression/self-perception (7 items), and embarrassment (3 items). The FIQL has shown to be reliable and valid [32]. A separate quality of life scale is included as FI is considered to highly influence patients' daily life since uncertainty exist on when incontinence episodes may occur.

▪ PREFAB-score: the PREFAB-score is a modified version of the widely used and accepted PRAFAB-score in the urinary incontinence field. The PRAFAB-score is a simple and quickly obtained score which summarizes several objective and subjective components of the severity of urinary incontinence complaints (ranging from 5–20) [33]. The score has proven to be reliable, valid and responsive [34]. Since most of the PRAFAB-score, except for the "amount" item, is applicable to fecally incontinent patients as well, it is suggested to adapt the PRAFAB-score to fecally incontinent patients. From the patients' point of view, two aspects about excretion of feces are reported to be highly embarrassing and affecting social life and body image: whether the excretion can be held up by a pad or not and whether excretion can be smelled by the environment or not. These two aspects, reflecting social limitations, are therefore integrated in the modified PRAFAB-score (min-max = 6–20; range = 15).

▪ Global perceived effect (GPE) (Table 2): this subjective score reflects the change in complaints after physiotherapy treatment compared to the period before treatment and ranges from 1–9 points. A score of 8 or 9 indicates much worsening of complaints and is an indication to consider co-interventions.

**Table 2:** Global perceived effect (GPE-score)

To which degree have your complaints changed compared to the period before physiotherapy treatment?
At the moment, the complaints are .....
1. Very much improved
2. Much improved
3. Moderately improved
4. Slightly improved
5. Unchanged
6. Slightly worse
7. Moderately worse
8. Much worse
9. Very much worse
..... compared to the period before physiotherapy treatment

Follow-up measurements are scheduled at 3, 6 and 12 months after inclusion. Primary outcome measure is the Vaizey score [28]. Success after physiotherapy is defined as a reduction in Vaizey score of more than four points (16.7%) compared to baseline measurement. Recently, it is reported that patients who rated their situation to be better, improved four points on the Vaizey score [31]. Secondary outcome measures are anorectal resting and squeeze pressure, rectal capacity, anorectal sensation, diary results, FIQL, PREFAB-score and GPE-score.

### Sample size

The sample size is calculated based on data from a recently published cohort study on pelvic floor rehabilitation in fecally incontinent patients [31]. Patients who reported their situation to be better after pelvic floor rehabilitation showed a mean improvement score of 4.33 points on the Vaizey score and patients who rated their situation to be equal or (much) worse showed a mean improvement score of one point (baseline average Vaizey score is 18). As subjective improvement can not be projected as decimals on the Vaizey score, we calculated two sample sizes: (1) with exact scores and (2) with absolute scores to correspond with the Vaizey score and estimate of the minimally important change (MIC). The absolute difference in improvement of four points between the patients who rated themselves to be better and the patients who rated themselves to be equal or (much) worse is considered the MIC on the Vaizey score between both trial arms [31].

The first sample size is calculated using an average Vaizey score of 13.67 and a standard deviation of 8.45 for sample 1 and an average Vaizey score of 17 and a standard deviation of 3.90 for sample 2, which resulted in a sample size of 106 patients. The second sample size is calculated using an average Vaizey score of 13 and a standard deviation of 8.45 for sample 1 and an average Vaizey score of 17 and a standard deviation of 3.90 for sample 2, which resulted in a sample size of 73 patients. The sample size is in a range of 73 to 106 patients and we decided to include at least 106 patients to feel confident about the required sample size.

All calculations assume a one-sided effect (first trial arm has a better outcome than second trial arm), an alpha of 0.05, a power of 80% and an expected drop-out of 10%. It is expected that the drop-out will be minimal since patients will be greatly motivated to participate and adhere to the protocol, by experiencing such an embarrassing and socially restricting disorder.

### Referral

After diagnosis and inclusion, patients are referred to an extramural private practice, preferably nearby their home address. Private practices are selected with physiotherapists experienced in women's health, who are registered at the Dutch Society for Physical Therapy in Pelvic Floor Disorders and Pre- and Postnatal Healthcare (NVFB). They are all educated and trained in the performance of invasive techniques, as is used during digital testing and RBT. In total, 90 physiotherapists participate throughout the Netherlands. The initial physiotherapy session should be within three weeks after referral. The patient hands over an acknowledged doctor's referral letter and fifteen rectal balloons (group 1) to the physiotherapist before the initial session. FI is not recognized as a chronic disorder and insurance coverage is patient dependent. However, physiotherapy in this trial is considered as being part of usual care, as included patients would also be excellent candidates for physiotherapy outside the scope of this trial. Since January 2006 a referral letter is not obligatory anymore.

### Physiotherapy intervention

Treatment is administered according to a standardized protocol, which has been developed by clinicians and physiotherapists specialized in the field of pelvic floor disorders. Physiotherapy consists of pelvic floor muscle training (PFMT) and rectal balloon training (RBT; only trial arm one). Patients receive 12 sessions within three months after the initial session, starting with two sessions a week during three weeks, and thereafter reducing to one session a week. The first session takes 45 minutes and all subsequent sessions 35 minutes. A case report form physiotherapy (CRF) is filled out for each physiotherapy session.

Physiotherapists are aware of details about the medical history, physical examination and diary, but blinded for findings of anal manometry, rectal capacity measurement, anorectal sensation, anal endosonography, defecography, FIQL and PREFAB-score. During physiotherapy sessions, information is obtained by performing peri-anal inspection, digital rectal evaluation and rectal balloon training (trial arm 1).

### Pelvic floor muscle training

PFMT is offered to all included patients and consists of selective voluntary contractions and relaxations of the pelvic floor muscles and the anal sphincter. Awareness of these muscles is necessary, as is the avoidance of the use of surrounding muscles like the adductors or abdominals. PFMT aims at maximizing strength, improving duration of strength, and improving timing and coordination of contractions. One treatment cycle starts with a series of 8–12 single maximum contractions, sustained for 1–3 seconds. During treatment the series are extended from 1–3, and the sustained maximum contraction from 1–3 seconds to 6–8 seconds. A one-minute rest is allowed between the cycles. One cycle is repeated three times. Furthermore, patients perform three sustained submaximal contractions of 30 seconds, with one-minute rest in between. Patients are also instructed to perform home exercises daily. At home the cycles and sustained contractions are done in the same way and three times daily. The exercises are practiced in different starting positions and circumstances, simulating as much as possible daily life. Patients' compliance is encouraged because the home exercises determine to a great extent the success of physiotherapy. Digital rectal examination is used to measure the ability of pelvic floor muscles to contract and to quantify the strength. A contraction is considered to be sufficient if the pelvic floor and anal sphincter lift inward.

### Rectal balloon training

A rectal balloon attached to a syringe is introduced in the rectum and slowly inflated with air to imitate rectal contents. Sensory threshold, urge sensation, and maximal tolerated volume are assessed. Patients with an insensitive rectum are trained to discriminate and respond to smaller rectal volumes of distension until a normal level of sensory threshold is reached or further improvement cannot be expected. In this way, patients receive an earlier warning from stool entering the rectum and impending defecation. In addition, patients can counteract reflex inhibition of the internal sphincter due to awareness of stool in the rectum [15]. Patients with a hypersensitive rectum are trained to tolerate larger volumes by the use of progressive distension and urge resistance until a normal level of urge sensation is reached or the urge does not diminish with time or is uncomfortable. Patients with weakness of the pelvic floor muscles learn to contract these muscles immediately and strongly in response to rectal filling.

### Details of each session

Details of each physiotherapy session are presented in Additional file 1: Details of each physiotherapy session.

### Co-interventions

After three months follow-up the physician evaluates the Vaizey score and the GPE-score. In case of an improvement of less than four points on the Vaizey score and/or a GPE-score of eight or nine, the clinician decides upon further therapeutic steps. If necessary, co-interventions are allowed after three months follow-up, although this should be avoided as much as possible. Examples of co-interventions are sacral nerve stimulation, anal repair or lavage. During physiotherapy treatment, patients are not allowed to use anal tampons.

The most common used drugs in patients with moderate to severe FI are Loperamid and sometimes Norit. Medical interventions and/or diet changes are first tried in the conservative management of patients with FI. If patients do not benefit from medical interventions, usual care implies referral for physiotherapy interventions. So, the majority of patients do not use drugs before physiotherapy management since they appeared not to be helpful. Nevertheless, patients who use drugs before starting with physiotherapy interventions are instructed to follow an unchanged stable medical regimen. Therefore, ongoing and change in medication use are monitored during the trial. In the analysis we will account for co-interventions, such as medication use.

### Data analysis

In case of longitudinal missing data, imputation is performed with the last observation carried forward method. Main outcome is tested according to the intention to treat principle. Both treatment groups are checked for comparability regarding baseline data. An independent t-test is done for continuous variables and a Chi-square test for categorical variables. Analyses are performed to compare differences within groups between baseline and short-term follow-up (3 months) and long term follow-up (6 and 12 months) regarding primary and secondary outcome measures. A subgroup analysis is done for the patients with and without co-interventions. Analysis of variance is used to determine the difference in mean change of outcome measure between clinical subgroups. A two-tailed p-value of 0.05 is considered to indicate statistical significance. Data analysis is done using SPSS version 13.0 (SPSS, Inc., Chicago, Illinois). Confounders that are taken into account are presence of urinary incontinence, use of anal tampons, compliance and medication use.

### Compliance

The ability of the patient to perform exercises at home is documented at the beginning of all physiotherapy sessions and if not, the reason is asked.

## Discussion

### Why publish a study protocol

It is decided to publish the study protocol before obtaining results as we think it is important to share our thoughts and decisions about designing this study with other researchers in the field. Furthermore, a research article offers only a limited space to explain and reflect on the methods of a trial. Finally, researchers in the field are aware of which study is ongoing, which prevents wasteful duplication of research effort.

### Bias and confounding

To minimize information bias, the performance of physiotherapists experienced in women's health is standardized. First, only physiotherapists registered at the NVFB are able to participate in this study. Registration approval indicates satisfying the outlined quality criteria by the NVFB. Second, participating physiotherapists receive a general and physiotherapy protocol of the PhysioFIT-study composed by experts in the field. Third, participating physiotherapists attend a general meeting. An individual visit is organised when a patient is referred to a non-attending physiotherapist. During the meeting, the physiotherapists were instructed on the general and physiotherapy protocol, a DVD-film illustrated the intended rectal balloon training and questions and discussions were raised. Some small adaptations were made in the protocol based on remarks from the physiotherapists.

To further minimize information bias, adaptations were made to the rectal balloons of the supplier. The current balloon reached the sigmoid colon, as soon as the balloon was filled with approximately 180 ml, possibly resulting in colic pains. This prevented the measurement of a higher maximal tolerated volume, although 180 ml is rarely reached. As this balloon was inadequate for this trial, adapted rectal therapy balloons were developed in cooperation with the supplier.

In this trial, it is impossible to blind on all levels. Most importantly, the measurement of the primary outcome is blinded as well as the outcome assessors. Blinded double data-entry is performed to assure correct data-entry.

Confounders that are taken into account are presence of urinary incontinence, use of anal tampons, patients' compliance and medication use. The presence of urinary incontinence can influence the perception of patients' quality of life and estimation of severity of FI, thereby distorting the scores on the FIQL, PREFAB and GPE-scores. Furthermore, patients' compliance to the home exercise program determines therapy intensity and indirectly therapy success. Therefore, the physiotherapist asks each session patients' compliance to the home exercise program as formulated. The patient gives an explanation in case of insufficient compliance. Finally, the use of anal tampons and medication use can influence the number of involuntary events and stool consistency, as measured during the visits to the physician and in the diary.

### Trial arms and patient selection

The effectiveness of rectal balloon training is assessed in a randomized controlled trial using two trial arms. A third trial arm with RBT as a single treatment option is not included, for the following reasons:

RBT, as a solitary therapy option, has another suggested goal than PFMT. PFMT is hypothesized to improve strength, coordination and endurance. RBT is hypothesized to teach patients with an insensitive rectum to discriminate and respond to smaller rectal volumes of distension until a normal level of sensory threshold is reached. Patients with a hypersensitive rectum are taught to tolerate larger volumes by the use of progressive distension and urge resistance until a normal level of urge sensation is reached. In our first trial arm RBT is performed to strengthen the effect of PFMT. To compare this trial arm with only PFMT, the additional effect of RBT can be assessed.

In addition, a review by Siv Mørkved, based on seven randomized controlled trials published between 1999 and 2005 on conservative treatment (PFMT with or without biofeedback and electrical stimulation) concluded that PFMT with and without biofeedback seems to be effective in reducing FI [35]. This shows us that many physiotherapy intervention trials are designed with PFMT being at least part of the intervention. Adding a trial arm enables us to assess the effect of a more optimized physiotherapy intervention. Based on this and consensus in patients with moderate to severe FI, it is decided that PFMT alone should be at least the minimum standard of care in this disabled patient group.

Finally, as a result of strict in- and exclusion criteria we expect to include only one patient a week, resulting in an unacceptable long inclusion and follow-up period or insufficient power when extending to three trial arms.

### Secondary goals

Secondary goals of the PhysioFIT-study are to assess the psychometric properties of the PREFAB-score in clinical practice. In addition, baseline measurements are checked for their ability to predict outcome of physiotherapy.

Furthermore, the responsiveness of the Vaizey score is reviewed. The Vaizey score is a more common used severity score in surgical trials. But less is known about the responsiveness of the Vaizey score in physiotherapy trials. One study with comparable in- and exclusion criteria and study population made a first attempt to correlate the Vaizey score with a Global perceived effect score (31). Patients with a decrease of 9 points rated their situation "much better" than before physiotherapy treatment, patients with a decrease of 4 points rated their situation "better" and the group that rated their situation "(much) worse or equal" had a decrease in 1 point (patients were asked in a standardized interview to score their current situation on a scale of 1 (much worse), 2 (worse), 3 (no change), 4 (better), or 5 (much better)). We decided that rating the patients' situation as "better" is a minimally important change (MIC), expressed in an absolute number and not in a certain percentage.

Next to the Vaizey score we included the subjective GPE score (range 1–9) in our trial as an additional outcome measure, as we believe it is important to further investigate the responsiveness of the Vaizey score in patients with FI in physiotherapy trials.

Finally, it is expected that the outcome after physiotherapy interventions is dependent on the baseline Vaizey score; therefore we will perform a stratified analysis on baseline severity scores and change scores.

### Transfer of knowledge and implementation

The results of this trial will be used for recommendations in the guideline of physiotherapy in fecally incontinent patients. This guideline will be developed according to method of clinical practice guideline development of the Royal Dutch Society for Physical Therapy (KNGF) [36].

### Spin off

Often, a trial is designed with the objective to make a difference in clinical daily practice. This section elaborates on what we believe might influence clinical practice.

Based on the results of this trial, baseline measurements are checked for their ability to be a prognostic factor for a good or poor outcome after physiotherapy. In this way, patients can be selected who benefit most of the proposed physiotherapy intervention. Furthermore, this trial can give information on the number of physiotherapy sessions after which more sessions are of no further benefit. Continuation or ending of therapy will have the same outcome at that point. Finally, if the effectiveness of a certain physiotherapy intervention is established, physicians can make a well-founded decision for referral to physiotherapy treatment, in stead of proceeding with surgical treatment. In conclusion, this trial might add to clearer referral patterns and better cooperation between physicians and physiotherapists.

In case of insufficient result of physiotherapy and proceeding to surgical treatment, the received physiotherapy intervention can still be of value. Patients already learned to be aware of their pelvic floor muscles and know how to contract them in an environment without post-surgical stress. Besides, their pelvic floor is probably in a better condition. Future research in this area can focus on the effect of different pre-operative physiotherapy interventions on the success of surgery in FI.

## Competing interests

The author(s) declare that they have no competing interests.

## Authors' contributions

EB substantially contributed to the design of the study and draft of the manuscript. BB, EH and RDB were involved in the design of the study, drafting the manuscript and revising it critically. CB and JM contributed to the design of the study, are involved in data collection and commented on drafts of the paper. WVG is involved in data collection and commented on drafts of the paper. All authors read and approved the final manuscript.

## Pre-publication history

The pre-publication history for this paper can be accessed here:



## Supplementary Material

Additional file 1 Details of each physiotherapy session. explanation of the content of each physiotherapy session within the PhysioFIT-study Click here for file
