# Supplementary material for: A randomized physiotherapy trial in patients with fecal incontinence: design of the PhysioFIT-study
Source: BMC Public Health. 2007 Dec 20;7:355. doi: 10.1186/1471-2458-7-355 (PMC2234416; doi:10.1186/1471-2458-7-355)
Supplement: Additional file 1 — Details of each physiotherapy session. explanation of the content of each physiotherapy session within the PhysioFIT-study [file 1471-2458-7-355-S1.doc]

Additional file 1: Details of each physiotherapy session

Session 1 (45 minutes)

- Explanation and information

In the first physiotherapy session, expectations of both the patient and therapist are discussed and the anatomy of the pelvic floor and anal sphincters is explained, as well as the functioning of the pelvic floor muscles. Furthermore, the patient receives details about the treatment program. Informing the patient will stimulate the compliance of the patient and improve the patient-therapist relationship [37]. For long-term success, the provision of training and information is important, especially with regard to pelvic floor use and toilet behavior, which should be incorporated into patients’ daily life.

Exercise therapy given in combination with the provision of information and training is an example
of a so-called behavioral technique, in which certain steps are important: the exchange of information and explanation (“thinking”), the awareness and feeling of the pelvic floor, posture and movement (“feeling”), exercising and training the pelvic floor (“doing”) and encouraging compliance to therapy over both the short and long term (“keep on doing”) [38].

- Explanation of pelvic floor muscle training (group 1 and 2) and rectal balloon training (group 1)

Session 2 (35 minutes)

- Inspection and digital rectal examination

Additional information for the physiotherapist is obtained by inspection and digital rectal examination. Inspection provides information about the perineal movement during contraction and straining of the pelvic floor. Digital rectal examination reveals pain complaints. Besides, pelvic floor muscle and external anal sphincter muscle strength is assessed according to the Oxford score, ranging from 0 (no muscle contraction) to 5 (strong contraction) [39]. Endurance of sub-maximal strength (maximum 30 sec) of these muscles and exhaustion of a maximum contraction (maximum 5 times 1 sec) is also determined. Finally, the sensory threshold, urge sensation, and maximal tolerated volume are assessed.

- Treatment according to treatment arm

Session 3 to 11 (35 minutes)

- Evaluation

-Discuss problems with the patient

-Evaluation by way of digital rectal examination to assess pelvic floor muscle and external anal sphincter muscle strength according to the Oxford score (treatment 9).

- Treatment according to treatment arm

Session 12 (35 minutes)

- Evaluation

-Discuss problems with the patient

-Evaluation by way of digital rectal examination to assess pelvic floor muscle and external anal sphincter muscle strength according to the Oxford score. In addition, endurance of sub-maximal strength (maximum 30 sec) of these muscles and exhaustion of a maximum contraction (maximum 5 times 1 sec) is determined.

- Treatment according to treatment arm
- Evaluation of treatment program and compliance to treatment program
